# “It is a challenge to do it the right way”: an interpretive description of caregivers’ experiences in caring for migrant patients in Northern Sweden

**DOI:** 10.1186/1472-6963-12-433

**Published:** 2012-11-29

**Authors:** Faustine Kyungu Nkulu Kalengayi, Anna-Karin Hurtig, Clas Ahlm, Beth Maina Ahlberg

**Affiliations:** 1 Department of Public Health and Clinical Medicine, Division of Epidemiology and Global Health, Umeå University, SE- 901 85, Umeå, Sweden; 2 Department of Clinical Microbiology, Division of Infectious Diseases, Umeå University, SE- 901 85, Umeå, Sweden; 3 Department of Women’s and Child Health, Uppsala University, SE- 751 85, Uppsala, Sweden; 4 Skaraborg Institute of Research and Development, SE-541 30, Skövde, Sweden

**Keywords:** Caregivers/caregiving, Culture/cultural competence, Immigrants/migrants, Interpretive description, Language/linguistics, Interpreters, Health care professionals, Religion, Thematic analysis, Sweden

## Abstract

**Background:**

Experiences from nations with population diversity show extensive evidence on the need for cultural and linguistic competence in health care. In Sweden, despite the increasing diversity, only few studies have focused on challenges in cross-cultural care. The aim of this study was to explore the perspectives and experiences of caregivers in caring for migrant patients in Northern Sweden in order to understand the challenges they face and generate knowledge that could inform clinical practice.

**Methods:**

We used an interpretive description approach, combining semi-structured interviews with 10 caregivers purposively selected and participant observation of patient-provider interactions in caring encounters. The interviews were transcribed and analyzed using thematic analysis approach. Field notes were also used to orient data collection and confirm or challenge the analysis.

**Results:**

We found complex and intertwined challenges as indicated in the three themes we present including: the sociocultural diversity, the language barrier and the challenges migrants face in navigating through the Swedish health care system. The caregivers described migrants as a heterogeneous group coming from different geographical areas with varied social, cultural and religious affiliations, migration histories and statuses, all of which influenced the health care encounter, whether providing or receiving. Participants also described language as a major barrier to effective provision and use of health services. Meanwhile, they expressed concern over the use of interpreters in the triad communication and over the difficulties encountered by migrants in navigating through the Swedish health care system.

**Conclusions:**

The study illuminates complex challenges facing health care providers caring for migrant populations and highlights the need for multifaceted approaches to improve the delivery and receipt of care. The policy implications of these challenges are discussed in relation to the need to (a) adapt care to the individual needs, (b) translate key documents and messages in formats and languages accessible and acceptable to migrants, (c) train interpreters and enhance caregivers’ contextual understanding of migrant groups and their needs, (d) and improve migrants’ health literacy through strategies such as community based educational outreach.

## Background

The proportion of international migrants has rapidly increased in the last few decades and this trend is unlikely to change in the near future [1,2]. While these transformations hold developmental potential for individuals as well as their societies of origin and destinations it also exacerbates existing problems and generates new challenges [1,3]. Likewise, immigration to Sweden has increased in recent decades, and now includes citizens from approximately 200 countries. The share of foreign-born which was only one percent of the Swedish population in 1940 increased to 14 percent in 2008 [4]. By 2060, one in five residents is expected to be a foreign born [5].

Evidence from the literature suggests that this continuous growth of foreign born populations with varied cultural traits and health profiles, presents complex challenges for health care delivery due to change in disease profiles, communication problems, diversity of cultures and institutional practices as well as individual past experiences and attributes [6-14]. These challenges are said to influence medical encounters and can result in mistrust that may lead to sub-optimal utilization of health services, frustrations and calamitous errors in diagnosis and treatment regimens [7-10,15,16]. Literature on cross-cultural health care indicates that migrant populations present unique challenges to health professionals, and can be a source of frustration for health professionals as they may struggle with uncertainty and apprehension when caring for these patients [6-13,15,17,18]. Previous studies suggest that caring for patients who speak a different language is difficult because of lack of or unavailability of interpreters or problems in accessing interpreter agencies, which in some cases force care providers to use family members as interpreters. Even when interpreters are used communication problems persist due to cultural differences and low professional status of interpreters [6,7,10,18,19].

Health care providers may also manage patients of diverse backgrounds in an unsatisfactory manner due to stereotypes, limited cultural awareness and ability, which can create barriers and resentment [6,8-11,14,15,18]. Some authors, however, acknowledge that health care professionals working among migrant communities are increasingly challenged to provide health care that is responsive to the special health needs of these populations [7,10,11,13-16]. Identifying potential challenges is thus important for successful healthcare delivery to these populations. In this article we present findings from a qualitative study on perspectives and experiences of providers caring for patients with migrant backgrounds.

### The Swedish health care system

The Swedish Health care system is decentralized and mainly tax-financed through counties and municipalities, although a small fee is charged for most services. Care for children and young people up to the age of 19 is free [20,21]. Despite a growing number of private care providers, up to 90% of health care is provided by the county councils and municipalities through the public care facilities, which include primary, hospital and public health and preventive care. The overall health care policy is under the responsibility of the state and health care is available to all legal residents who can access it by registering with the nearest primary health care center in the vicinity of their home or a private provider of their choice [20,21]. All legal residents are allocated a personal number by the tax-authorities which is used to access all public services including health services [21]. Primary care is the basis of health and medical care in Sweden. Primary care staff consist of doctors, nurses and other staff who work together to provide an extended range of services from general practitioner care to physiotherapy and counseling. Nurses have a greater role in health care as they have specialized competence in treating long term conditions such as asthma, diabetes etc…[20,21]. Except for emergency cases when patients are advised to call for ambulance or to present themselves to the hospital emergency room, non-emergency situations are dealt with at the local/primary health center or private clinics. Patients are encouraged to call the local health center or book an appointment through the phone or on the web, but surgery staff answers the phone calls only for restricted periods. The local health center may refer patients to specialists at the hospital or private clinics when needed [20]. According to the Swedish legislation, care providers have the obligation to reinforce the position of the patient. For instance, they have to provide individually tailored information and guarantee the patient the freedom to choose between treatment options and the right to a second opinion in life threatening or other serious diseases or injuries [20,22]. Usually, the patient gives an oral or written consent after consultation with the staff. Most often it is enough that the staff concludes that there is consent or it is implied through the patient actions (e.g. facilitate the action to be initiated) [22]. The Swedish health care system is also well known for its use of distant communication technology such as various electronic reply and call-back systems, electronic prescriptions and web booking systems.

### Migrants’ access to the Swedish health services

All legal migrants are entitled to care on the same terms as other Swedish residents. Asylum seekers are entitled to only care that “cannot wait” in accordance with the law on health care for asylum seekers [21,23]. When they seek care they are required to show a special personal card known as “LMA (*Lag om Mottagande av Asylsökande*: Law on the reception of asylum seekers) card” provided by the migration authorities. The care they are entitled to include maternity care, care in relation to abortion and family planning. Non-residents and other unofficial migrants may also access care services but, they have to pay the full cost [21]. By defining an immigrant as a foreign-born person, legally admitted and expected to stay at least 12 months in Sweden, Statistics Sweden excludes asylum seekers and undocumented migrants [4]. Therefore, we did not adopt Statistics Sweden’s definition in this study. Instead, we use the term “migrant” to refer to all foreign-born persons living in Sweden.

Except for emergencies, the first contact of migrants with the Swedish care system usually takes place during medical screening, offered especially to migrants from countries with high prevalence of infectious diseases such as HIV/AIDS, tuberculosis, hepatitis and other sexually transmitted infections (STIs), and this is in accordance with the Swedish social welfare regulations and the Swedish Communicable Disease Act [24]. All newly arrived migrants from selected countries receive a letter from local health providers in the vicinity of their residence inviting them to undergo medical screening [24]. After this first contact, migrants are expected to make contact and interact with health care providers like other Swedish residents when they need care. Despite the recommendation to offer language assistance and use interpreters when caring for patients who do not speak or understand Swedish, studies have shown that care providers still face complex challenges identifying and meeting the basic needs of patients from culturally diverse populations [6,7,10,19,25,26]. According to Szczepura, in countries and regions with diverse population, there is extensive evidence on the need for cultural and linguistic competence in health care organizations [10]. Szczepura described linguistic competence as the capacity of an organization and its staff to communicate effectively, and convey information in a manner easily understood by diverse audiences including persons of limited language proficiency, and those who have low literacy skills or are not literate [14]. Betancourt et al., defined cultural competence in health care as the ability of systems to provide care to patients with diverse values, beliefs and behaviors, including tailoring delivery to meet patients’ social, cultural, and linguistic needs [27]. In Sweden, despite the increasing cultural and linguistic diversity only few studies have focused on challenges in cross-cultural health care [6-8,10,11,19,26]. Additional research is thus needed in this area.

### Aim

The aim of this study was to explore the perspectives and experiences of healthcare professionals in caring for migrant patients in order to understand the challenges they face and generate knowledge that can inform policy and clinical practice.

### Conceptual framework

In an attempt to explain the experiences of care providers in caring for migrants living in Sweden, we found relevant the socio-ecological model (SEM) because of its focus on the relevance of social contexts and the intertwined relationship between individuals and their social environment (community norms and values, regulations and policies) [28,29]. Additionally, the model suggests that factors at different levels may not only impact on individual behavior, but may also impact on or modify each other [18,29]. The rationale for our use of the model is to address the factors influencing health care delivery for migrants in Sweden. Other authors used the model to investigate disparities and barriers to accessing health services, and intervention opportunities for ethnic minorities and migrant populations [18,30]. We adapted the model to describe the impact individual, interpersonal, institutional, and the societal factors have on the delivery of care to patients with migrant backgrounds, the diasporic tensions and the ways migrants negotiate the new and old world. More specifically, we used the model here to shed light on the experiences of care professionals caring for patients with migrant backgrounds.

## Methods

### Study design

We were guided by the need to explore the perspectives and experiences of caregivers in caring for migrants, issues stemming from these experiences and the need to understand and generate knowledge that could inform clinical practice. We decided to combine interviewing and observation in an emerging design using the interpretive description (ID) approach described by Thorne and others as the sensitizing framework for the study [31-33]. Additionally, we included participants with varied professional backgrounds to get a better understanding and broader view of care providers’ experiences and thus enhance credibility [32-35]. Thorne and colleagues described ID as non-categorical methodology that emerged in response to a call for an alternative way of generating grounded knowledge relating to clinical practice and that aimed at moving qualitative inquiry to a more abstract form of interpretation beyond the level of description [33]. The ID approach acknowledges the researcher’s theoretical and practical foreknowledge of the phenomenon under study. Moreover, detailed line-by-line coding is avoided in favor of asking broad questions [31-33].

### Study setting and participants

A total of ten health professionals were purposefully recruited by the first author (FKNK) to represent different professions and varied backgrounds. FKNK contacted each potential participant by e-mail that also included information about the study, that participation was voluntary and that the informed consent form attached to the message would be collected on the day of the interview. The only criterion for selection was that they often come into contact with foreign-born persons in their daily clinical practice and are therefore experienced in caring for migrant patients. FKNK identified participants while working as an in house interpreter in Northern Sweden and interviewed the 10 recruited participants at primary health care centers, hospital clinics or County council after agreeing to be interviewed. As a qualitative study, sample size was evaluated on an ongoing basis to identify when saturation was reached [32,33]. The ten participants were predominantly Swedish (n = 8), one had a mixed background (half Swedish half other European decent) and one had non-European background. Six worked at a hospital, two at a health care center in a predominantly immigrant area and two at the county council. Except for one participant who preferred to be interviewed in English, all participants were interviewed in Swedish. Table 1 shows the study participants by profession, gender and specialty.

**Table 1:** **Study participants by profession**, **gender and specialty**

**Profession** (Identifier)	**Gender**	**Specialty**	**Number**
Nurse (N)	Women	District Nurse	1
		HIV Specialist Nurse	1
Social Worker (SW)	Women	HIV(Human Immunodeficiency Virus) and STIs (Sexually Transmitted Infections) Counseling	2
Doctor (D)	Man/Woman	Infectious Disease Specialist	2
	Woman	Pediatrician	1
	Man	General Practitioner	1
Public Health Officer (PHO)	Man	Infectious Disease Control	1
	Woman	HIV/STIs Prevention	1

### Data collection methods

#### Semi-structured interviews

We used a series of face-to-face semi-structured interviews as our primary data source. FKNK conducted the interviews between March 2009 and April 2010, using an interview guide developed based on findings from a previous survey study on knowledge and attitudes towards HIV/AIDS and tuberculosis among migrants attending language schools. The experience of the first author as an interpreter was particularly useful here [36,37]. A pilot study with 5 care providers working at the hospital (2) and two primary health care centers (3) was carried out in the study setting to evaluate the interview guide which resulted in some questions being rephrased. The interview guide was also refined throughout the process on the basis of emerging issues and observations. The following were the main issues in interviews: views on the situation of migrants in Sweden, challenges encountered when caring for migrant patients, personal experiences working with interpreters; barriers to the use of available health services by migrants; contexts when discrimination is likely to occur and providers’ views on migrants’ knowledge about diseases such as HIV/AIDS, tuberculosis and hepatitis. There was no need to continue interviewing after the ten interviews as similar responses were repeated and saturation was reached.

The audio-taped interviews took place at the participants’ offices or another convenient location and lasted for 1 to 2 hours. After each interview, the first author wrote supplementary field notes, transcribed the taped interviews and read through the transcripts to allow insights emerging to be incorporated in the ongoing data collection [31-33]. For instance, religious beliefs and gender norms and the way they impacted on the encounters were observed early in the interviews and were followed in subsequent interviews.

#### Participant observation and the position of principal investigator

We used participant observation as another method of data collection. FKNK who is fluent in five different languages had worked as a trained interpreter within the health care setting since 2005, before becoming the main investigator for the current inquiry by the end of 2008 when ethical approval was obtained. Working as an interpreter provided the main investigator with an opportunity to enter the field, observe and participate in many care encounters, thus becoming a participant observer. During her duties as in house/phone interpreter, she could learn by observing or hearing the way migrants and care providers were interacting during the encounters and identify/notice incidents in different care settings as they were happening. FKNK was not participating as a regular worker within any of these settings, but simply making a number of key observations on the encounter. The observation material thus consists of reflective memos of the first author’s professional experience as an interpreter and as a medical doctor with a master’s degree in public health. She shared a professional background with providers and a cultural background with patients as a migrant. Obviously, she conducted this research not only in her role as an academic researcher but also in her role as a migrant, a cultural mediator and health professional. All these roles might influence and shape her way of being in terms of the assumptions and biases that she brought to the study. Nevertheless, her perspective is also shaped by her position as a researcher including literature used and academic conferences on the research topic as well as reflexivity that enables researchers to reflect on and to critically question their assumptions and to deal with biases [38].

### Data analysis

We used thematic analysis approach and followed different phases involved in the approach as described by Braun et al. [39]. The analysis started with the first author transcribing, reading and re- reading the data (familiarization with the data). This was followed by a coding phase where the first and the last authors both with migrant backgrounds read the transcripts separately to identify patterns and linkages in the data and labeled paragraphs that contained information relating to each particular point being made. The codes were refined in ways described by other scholars [31-33,40]. Two other Swedish members of the research team, the second author, a senior public health researcher within the field of health care systems and the third author a senior consultant in infectious diseases also read the interviews, participated in the analysis and acted as points of reference for issues within the Swedish health care system. In the later phase of searching for themes, we compared and discussed the meaning of codes and emerging patterns to reach agreement and collate codes into potential themes following the process in thematic analysis [39].

**Figure 1:**
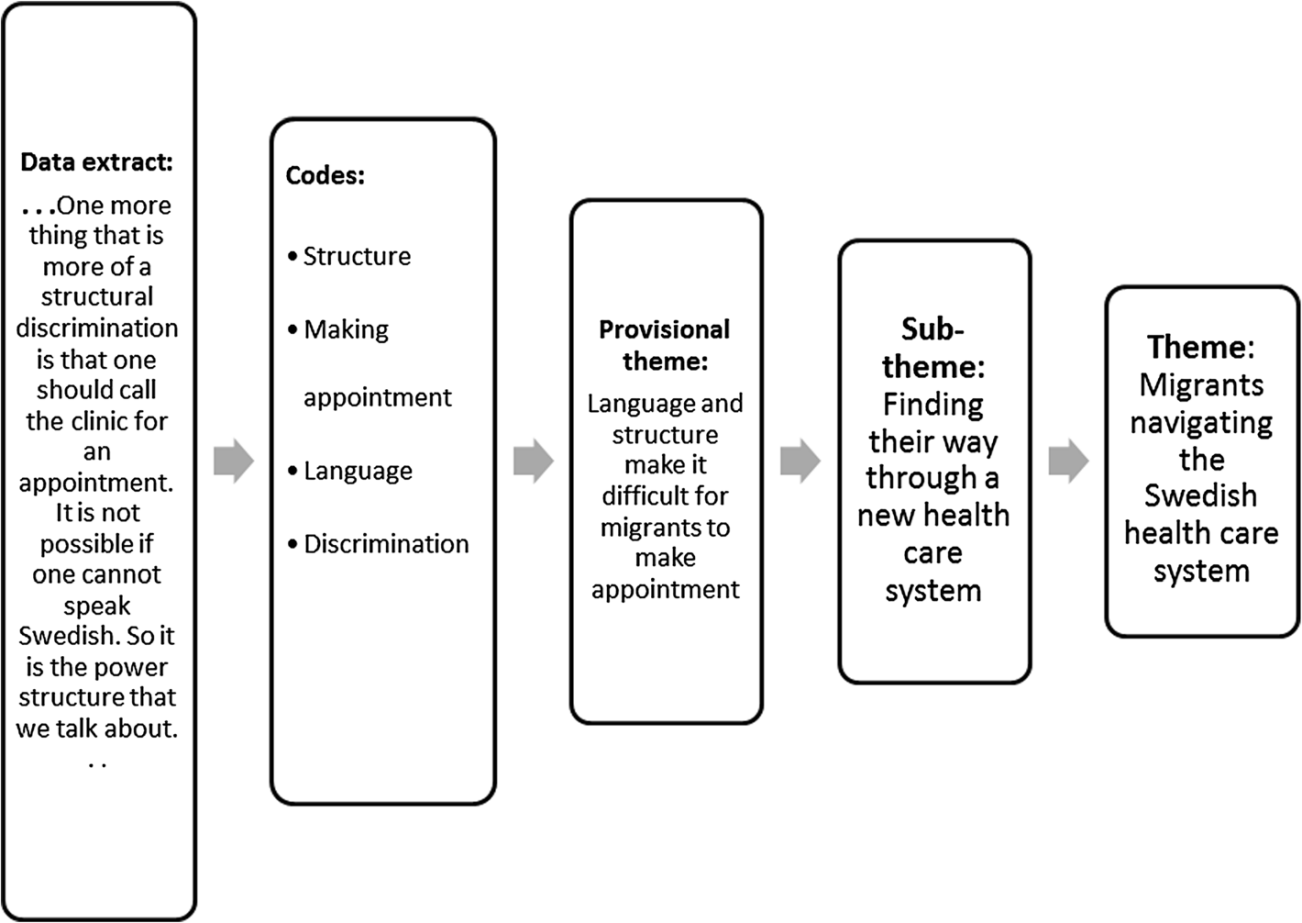
**A description of an example of analysis steps.** Example of data extract from interviews, with codes applied, initial theme, sub-theme and developed theme.

**Figure 2:**
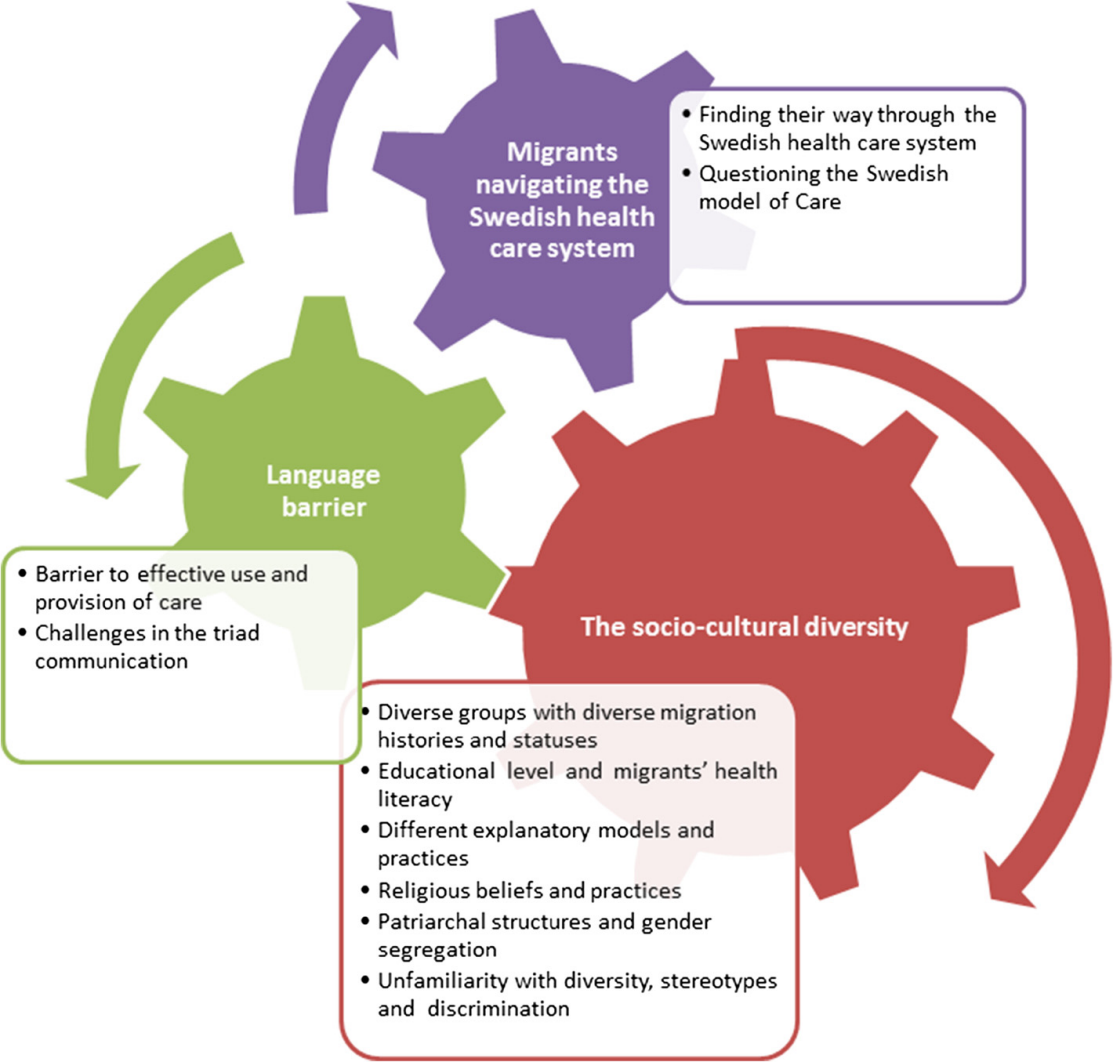
**A final thematic map**, **showing the final three main themes and sub**-**themes.** The arrows show the interconnection among themes and sub-themes.

Conceptual themes were inductively derived from analysis among and between individual interviews [31-33]. Our thematic analysis was guided by the analytic question: What challenges occur in the cross-cultural care encounters? While answering this question, we identified challenges for care providers as well as for migrant patients. The phase 4 of reviewing themes was guided by the socio-ecological model; and consisted in identifying factors at different levels of the model that were said to affect care delivery and receipt, and the medical encounter. Thereafter we developed a preliminary analytic structure from the provisional themes to form the basis for identification and explorations of commonalities and differences among and between the experiences of participants [18,28,29,31-33]. Observation notes were also used to scrutinize the provisional findings and to enhance trustworthiness. Figure 1 represents an example of analysis steps with data extract, codes, provisional theme, sub-theme and theme. Finally, in this process, we developed the following three interconnected themes that we present in this article: a) socio-cultural diversity b) language barrier c) migrants navigating the Swedish health care system. Each theme contains sub-themes pertaining to factors that were considered to affect care delivery and receipt, and medical encounter as illustrated by Figure 2. Within each of the sub-themes we explored the ways these factors affect care providers' experiences.

### Ethical considerations

The regional ethical committee at Umeå University approved the research project and the study was conducted in accordance with the Helsinki declaration [41]. We obtained both oral and written informed consent prior to all interviews. To protect confidentiality, we removed all names and other identifiers from transcripts and records, and we presented the results anonymously and limited access to the data only to the research team.

## Results

**Figure 3:**
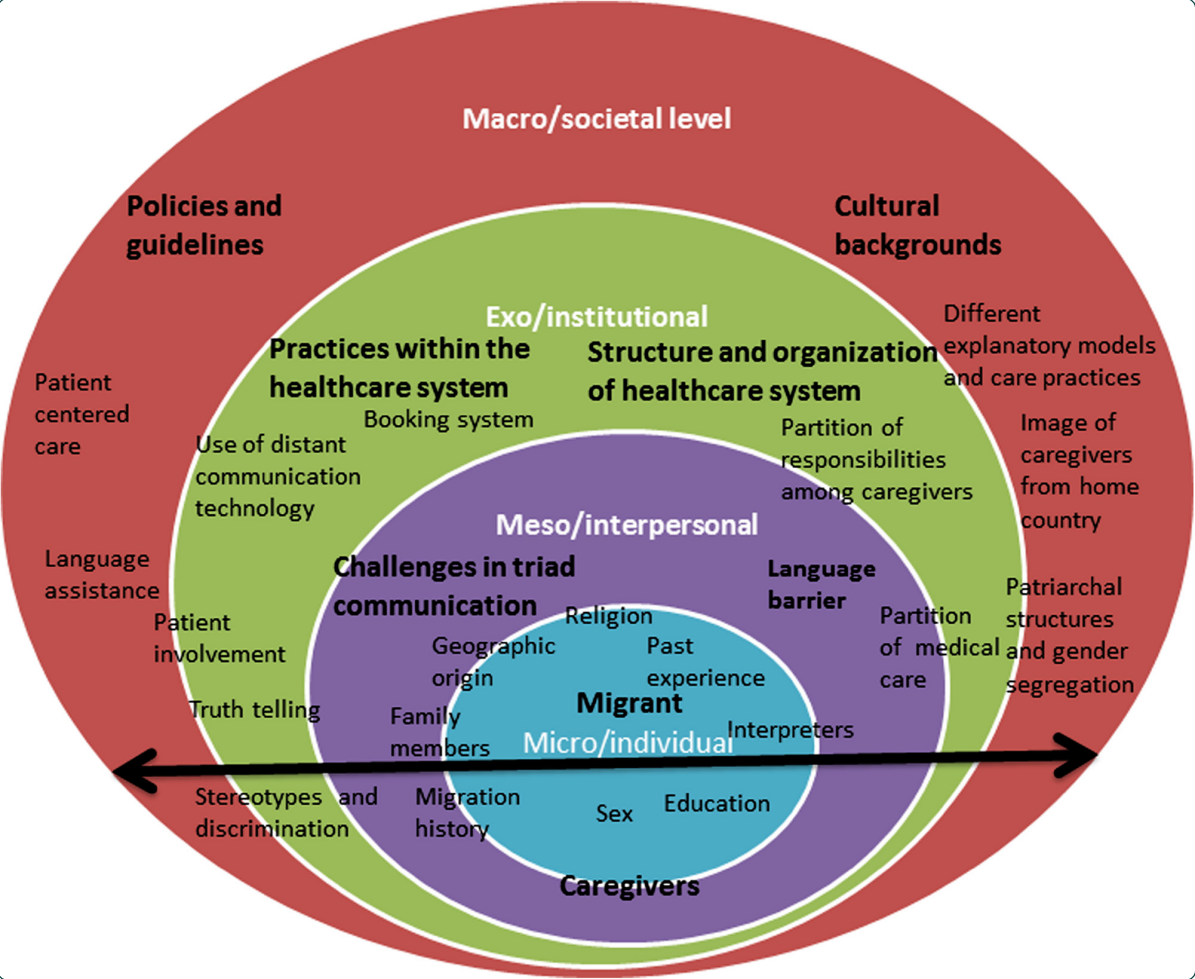
**A socioecological model of challenges in caring for migrants.** The arrow extending throughout the four levels suggests that factors or challenges at different levels extend into and interact with each other.

In this section, we present the various ways the care providers described their experiences in caring for migrant patients. According to the health care providers, migration history and status, educational level, language, cultural background and past experience with health care play an important role in access and delivery of care for migrant patients. Within each theme, we identified factors at different levels of SEM that caregivers described as challenges. The influences of cultural factors including health beliefs and practices, family dynamics, gender norms and past experience with health care services that were described as having an impact on care encounters. Furthermore, health care policies such as providing language assistance and strengthening the patient position (patient centered care approach) were also reported to directly or indirectly influence migrants’ care seeking behaviors, the delivery of care and medical encounters. The structure and organization of the health care system, different practices within the health care setting such as different roles of caregivers, booking system, the use of distant communication technology are institutional factors said to impact on access and provision of care. Language barrier and religious interferences in the relationships among caregivers, interpreters, migrants and their family members negatively or positively affect the encounter by the way they interact with each other and impact on provision and receipt of care. Finally individual factors such as age, sex, education, geographic origin, knowledge and attitudes for both migrants and care providers were also said to affect care delivery and receipt and the interactions during the encounter. Figure 3 illustrates an ecological model of challenges described by caregivers. Although each factor is only listed under one level of the system their influences exert on and interact with other factors at different levels. The following section highlights the ways these factors were said to affect care delivery and receipt as well as the encounters between caregivers and patients in question.

### The socio-cultural diversity

The care providers described both elements that relate to the individual which include education, gender, migration histories and religious beliefs of migrants as well as attitudes of providers towards migrants, and cultural factors including family dynamics, gender norms, health beliefs and practices all which were said to influence the interaction between migrants and providers and care encounters in the ways elaborated below.

#### Diverse groups with diverse migration histories and statuses

The health care providers interviewed described migrants as a heterogeneous group coming from different geographical areas with different social, cultural and religious affiliations, migration histories and statuses all which influenced the health care encounter, whether providing or receiving. One Swedish doctor described this diversity in the following manner while giving her opinion about the general situation of migrants in Sweden:

"It's such a huge diverse group, I think. It's really hard you can’t just say immigrants. They're like really, really different. Both how one gets here: if one has permission to stay, if one is an asylum seeker, if one is underground, if one is properly trained or if one is not so well educated, if one has had the opportunity to attend school or has not had the opportunity, if one gets a job or not, where one lives. I mean it’s a hundred different things that affect how one has it when one lives here. It's really hard, and how old one is. All matter. . . . However, it is also very different, if one is well educated and come from a country not located near Sweden, the difference is huge. (D_1_)"

However, not all staff members are aware of the heterogeneity. From observations by FKNK some staff especially those working at reception desks were not aware of the legal status of the foreign-born persons or the care they are entitled to. They seemed to have difficulties distinguishing a refugee from an asylum seeker. For instance, two cases were observed where the statuses were confused. The first concerns, a quota refugee (a person who gets protection via the United Nation High Commissioner for Refugees and receives the Swedish residence permits within the framework of a special refugee quota set by the Swedish government). This group has right to health care but the particular person was asked to show his LMA card which most asylum seekers carry as evidence that they are registered at one of the migration board regional offices and are awaiting decision to stay or to leave. The second case is about foreign born residents from Non-Nordic countries who have moved to Sweden for family ties. They too were asked to pay as the non-residents or visitors who are not entitled to care in Sweden while they were waiting for their “*personnummer*” (personal identity number) from the tax-authorities despite having residence permits which guaranteed them access to health care.

#### Educational level and migrants’ health literacy

Education was said to affect knowledge, views and perceptions of migrants on clinical realities. Highly educated migrants were said to be knowledgeable about the body and certain diseases and could thus understand even complex situations. In contrast, those with low education had not only poor knowledge but also problems to access, process and use available information. Giving information to this heterogeneous group was described as challenging because most of the time it was difficult to determine exactly how much they knew or what they needed to know. Dominant issues mentioned were lack of knowledge about the human body and how it functions. A Swedish doctor said:

"It is challenging to provide information in a good way, to know what level. . . . I ….often suspect that they perhaps do not know much about human body, how it functions. And some perhaps know a lot about it but it's hard to know how much. (D_4_)"

Explaining asymptomatic diseases (whether chronic or infectious) to low educated patients was described as particularly challenging because those with limited knowledge do not understand the connection between an infection and a disease, and in the absence of symptoms, they hardly believe they carry a virus that could be transmitted. This might according to the participants, unconsciously get them into legal problems for exposing others to the risk of HIV. A public health officer explained:

"If one has a low level of education one cannot easily understand that one has a virus when one is not sick . . . that one is infectious without symptoms. . . . So that was what happened to this person who was forced into isolation. He could not quite understand that he was infectious. He had no symptoms. (PHO_1_)"

A social worker elaborated further that the language used to tell diagnosis to low educated migrants with poor knowledge of diseases can also confuse them, leading to misunderstandings as the quote below indicates:

". . . So you have to find out what they know about the infection. I mean, I have experienced myself a problem, it was the language problem where a patient did not know what HIV was, but he knew what AIDS was and that there was an AIDS virus something like that and it became so obvious that the person had no idea about a person being HIV infected…. we are using that word. This is what you have to find out: what does the person know, what is the virus, what is infectious. . . (SW2)"

Lack of education was expressed as a barrier to access available information. This did not however stop some caregivers providing migrants with written information which was useless or referring them to websites without checking their literacy skills as was observed by the first author. Some migrants were for example, informed that they would receive their test results by regular mail and some received pamphlets or were advised to find information about their conditions or self-care material on official websites.

#### Patriarchal structures and gender segregation

The caregivers described their frustrations over what they viewed as patriarchal cultural practices that make men consider themselves as the primary authorities for giving/receiving medical information about other family members and protecting them, thus challenging the Swedish guidelines which stipulate communicating exclusively with the patient. The caregivers described their inability to cope with men who involve themselves in all aspects of care of their family members. One doctor said:

"So it's like the man has the responsibility to know and the rest of the family do not. One should not tell diagnosis, especially if it is a scaring diagnosis because the man has the duty to protect the rest of the family from this as well and then you have to somehow justify that you actually need to inform everyone but it is not easy to know how to justify it, I think it is really hard. (D_1_)"

Likewise, the first author observed such practices with men wanting to act as interpreters for their wives or to be present during care encounters. In one case, a woman who needed contraceptives waited until her husband left the room to whisper to the interpreter (first author) to inform the provider in confidence that she needed contraceptives to avoid having more babies. A public health officer also described gender discrimination/segregation in access to health information. She explained that the men (migrants) who are overrepresented among health educators within migrant associations talk exclusively to boys and the girls are left out. She said:

"…. They have often boys that they talk to … and it's okay to talk to boys on condom in a different way than with the girls, and especially if you have a religious background … But, with the girls it’s a bit like … One does not want to talk to them about … I feel that the girls are a bit left out somehow… (PHO_2_)"

A striking corollary of gender norms mentioned was patients’ request for providers of the same sex. Here the issues of religion and gender were intertwined and were lifted again by caregivers who described how they struggled to care for patients of opposite sex. The male doctor with migrant background told about his difficulty in caring for women patients in this way:

"Perhaps because of religious beliefs, people want either a man or a woman doctor. This is also something that we encounter. . . . If you have a woman who comes to you and she is hostile to men doctors, it does not really work well as it should. You lose trust, you can’t communicate. (D_2)_"

In contrast, two female Swedes, a doctor and a mature social worker explained that gender had never been an obstacle to them when dealing with migrant patients. The female doctor argued that she had never felt uncomfortable while dealing with male patients and the social worker was talking on account of her age as an advantage to deal with male patients and stated:

"… But, one should not have prejudices that men and women cannot talk about sex with each other. It would be dreadful. I still think about it sometimes, anyway … but I think at that age I am today can be an advantage… (SW2)"

#### Religious beliefs and practices

The caregivers also described how frustrated and powerless they felt over religious practices and belief in super natural powers, fatalism, fasting, travelling back home for religious rituals and refusing drugs assumed to contain religiously forbidden ingredients. All these in one way or the other were said to interfere with care given. One doctor explained how hard it was to understand that so many believed in God’s healing power, but at the same time needed medical evidence to prove it:

"I mean believing that "God will heal me.”. . . I also understand that they would pray for her, what I do not understand is why they should have a certificate where it is written that “I have HIV with cell T4 value. . . .” It is against my way of thinking. If it is God who wants to know it anyway, God would be able to keep track of it. (D_3_)"

The caregivers explained additionally that in some cases religious practices involved travelling back home for rituals, thus discontinuing the treatment. Two caregivers expressed frustration over fasting, especially when migrants were unwell or needed to combine medication with a meal at specific hours. A nurse explained:

"We have several patients who fast. For me it's a bit strange because some are not fully healthy. Then it’s a bit hard to understand that they have to be fasting until the sun goes down. . . . But, I understand that religion is very important. (N_2_)"

Another way in which religious beliefs interfere with care was described by an immigrant doctor who said:

"One perhaps suspects that maybe the drug is made of, or contains certain things that the religion prohibits. For example, my patients said they did not want drugs such as those for stomach. They think it contains something from the pig then you understand why people refuse. (D_2_)"

#### Different health explanatory models and care practices

The care providers reported encountering problems in understanding and explaining things to patients who hold different explanatory models about clinical realities. One doctor explained:

"Then it is obviously a challenge to properly understand what the patient says. . . . You sometimes have slightly different views on what is illness and what is not disease. So, sometimes you can get it all wrong and misunderstand. Then one should realize that it is so and ask further: did I understand right? What do you mean? (D_1_)"

For the migrant doctor, it seemed that decision making around an illness back home and the organization of care altogether influenced health seeking behavior:

"Then perhaps it is not a tradition in the home country to seek medical care in the first place, maybe it is parents or family members or the relatives who take care of each other before they seek care. Then something I notice is that people seek care at a very late stage. They’re waiting and thinking that it will go over. Then when they finally decide to seek care they are already very sick. (D_2_)"

He added that migrants who are accustomed to viewing illness in terms of symptoms seek treatment only for existing symptoms without regard that other underlying diseases/causes also need to be checked. A Swedish doctor added that the symptom driven health-seeking behavior illustrated that migrants did not fully understand health promotion initiatives:

"We deal with health problems before one feels sick. It's something that is very difficult to take in when one comes from another culture or different circumstances. . . . I mean one deals with the problem only when it occurs, right? (D_3_)"

#### Unfamiliarity with diversity, stereotypes and discriminatory attitudes

The participants expressed the view that certain behaviors and attitudes by the caregivers towards migrants perpetuated mistrust and fear of discrimination. Some were critical of their colleagues whom they described as lacking not only knowledge about migrants’ social, political and cultural backgrounds, but also skills to deal with patients of diverse backgrounds. This often resulted in stereotypes and negative attitudes. A nurse explained:

"Some nursing staff can discriminate people because of where they come from. And it’s because they don’t know or they haven’t got any good knowledge about people. There is a form of discrimination. . . . They can be or are quite racist. It is not just to the ones from the African continent but it’s to every form of immigrants. They don’t just like things that are different. (N_1_)"

When asked how discrimination could occur, one Swedish doctor (D_3_) hesitated before replying: “we certainly all have prejudices. Of course, we all use expressions that are prejudicial and Swedes are not the first.” Another explained that some caregivers had preconceived assumptions of migrants’ sociocultural backgrounds and tended to stereotype them:

"I think the problem is that one considers these as strange people. . . . One assumes somehow that these are people who come from a country in war, who have no education. There are a lot of prejudices and stereotypes. (D_1_)"

### Language barrier

This section focuses on the way language was said to affect care encounters. Language was reportedly complicated by institutional elements within the Swedish health care system such as use of distant technology to communicate with patients.

#### Barrier to effective provision and use of health care services

Participants described language as a major barrier in the care encounters. One view expressed was that language difficulties caused undue delay in establishing contact and acquaintance with patients and generated communication problems. Furthermore, explaining diagnosis and treatment was difficult and assisting those in need of attention was often impossible because of language as one nurse expressed:

"And it feels disappointing not to be able to be there as support for them and if one cannot speak the language, then it’s difficult for a patient to call and say I feel really bad. . . . Then one can also feel it that sometimes it takes a bit longer to get the contact. (SW1)."

Language was said to prevent migrants from accessing available services largely because information was provided in Swedish. They were also said to have difficulties making contacts with caregivers when in need especially through the phone, the usual practice in the Swedish health care system. One doctor explained: “… language is often a substantial barrier, I think. It's hard to find your way through the Swedish health care system when you do not master the Swedish language (D_4_)”. Caregivers explained that the inability to read and comprehend correspondences written in Swedish led to delay or failure to attend important appointments resulting in non-attendance fine. The migrant doctor exemplified it in the following quote:

". . . Another thing about migrants is that sometimes they do not really understand. Maybe you say in the morning, they understand it is the afternoon. They come later in the afternoon and say “I have an appointment with you.” (D_2_)"

The first author similarly observed the non-attendance problem when she was booked to interpret and in some occasions the patient did not turn up at all.

#### Challenges in the triad communication

Although they acknowledged that interpreters played a crucial role, the caregivers were concerned about inaccurate translations, patient discomfort, unprofessional interpreters and inability to prevent friends and relatives from interpreting. The following quote exemplifies inaccurate translation:

"I have been sitting and talking about measles and I heard that they said: “tuberculosis” and other things. Then one has to step in and say: “I've been talking about measles.” ‘But, I do not know what it's called in my language.’ “Then do not say anything.” (D_1_)"

The social worker emphasized the difficulty to communicate through an interpreter as patients were reluctant to talk openly with a third person sitting in the room. She explained:

". . . Sometimes I have met the patient for quite a long time and I understand that there is something behind, something that makes their anxiety strong, but that does not come out then, while we have an interpreter and I think I still feel that I know the patient pretty good. Then the first conversation without an interpreter, so It’s just like puff! Everything comes out or when the interpreter has gone or when I hang up the phone. . . (SW1)"

Caregivers also reported that migrants particularly those with low education do not access important information, as they are dependent on others to read letters or medical instructions from caregivers. In some cases, this was said to result in accidental disclosure of stigmatized conditions which can be debilitating. A nurse explained:

"It's very difficult for those who are illiterate. Apart from that they come from another country, they cannot even read or write in their native languages then it is more difficult for them even here to access information. . . . They are dependent on someone else who read for them. . . . . And they become dependent in so many different ways. (N_2_)"

Accounts of migrants desperately in tears were also common. The first author for example, witnessed patients complaining to their caregivers about disclosure of their HIV status when trying to get assistance from friends, relatives or roommates to read letters from caregivers, resulting in stigmatization and discrimination.

### Migrants navigating the Swedish health care system

In this section, the caregivers elaborate on how migrants’ past experiences with health care; the structure and organization of the Swedish health care system particularly the use of distant communication technology; and the patient centered care policy all affect the encounter between migrant patients and care providers.

#### Finding the way through a new health care system

Migrants were mostly considered to be unfamiliar with the organization of the health care system in Sweden or to have little knowledge of how it works. A doctor described the difficulty for migrants to navigate through the Swedish health care system in this way:

"It is a bit difficult to get around. . . how it works, for example, the hospital, the health center, when you send a referral to another clinic here at the hospital that they are then put on a waiting list and later on they will get a letter that will tell them that they have an appointment. (D_4_)"

The use of communication technology was said to affect the ability of migrants to access available services. Apart from language difficulties, some migrants were said to lack skills to access educational and self-care material, to book or cancel appointments or communicate with caregivers through the phone, websites or e-mails. Consequently, they sought care as drop in patients and it was difficult to convince them they needed an appointment ahead. The caregivers talked of this as a form of structural discrimination that could prevent migrants using available services, resulting in frustration and feeling of being discriminated against as explained below:

"One more thing that is more of a structural discrimination is that one should call the clinic for an appointment. It is not possible if one cannot speak Swedish. So it is the power structure that we talk about. (D_1)_"

The migrant doctor corroborated this opinion by saying:

"Sure, if you look at the Swedish families, most or nearly 90 percent have e-mails and are able to go online and cancel their appointments. You can do it on your mobile phone with voice mail. Not all immigrants can have access to it, know or understand that it can be cancelled. (D_2_)"

The caregivers described some migrant groups as impatient, very demanding and sometimes aggressive. They require immediate care or prescriptions even when it was considered unnecessary as described by this nurse:

"Sometimes you can see that it is nothing acute for today. . . . We have a few patients who are very demanding and can become quite aggressive. They always want to see a doctor. . . . They get angry if they can’t get to see that doctor. (N_1_)"

A social worker also described how some migrants almost give orders for the care to be provided or to get what they needed:

"… And this is mainly migrants from certain countries. That you can feel like as it is an order, "I should have, I should have, you should fix, you should arrange". It feels like they can almost the book of laws about their rights before they come to Sweden. (SW1)"

According to the migrant doctor, the lack of patience is due to limited experience and information about the Swedish health care system or the differential systems of care. He argued that what migrants sometimes perceived as discrimination might simply be their experiences of a different health care system:

"Most immigrant patients want help right away and they want it to happen quickly. . . . Because it is not everyone who understands how the medical care works. There is also a very difficult issue; someone who feels discriminated against, perhaps it is not actually discrimination. . . . It is also because the encounter does not work. They feel misunderstood and then they also think it takes too long to meet the doctor. It is also true when one thinks of some countries where you order all the tests at once and it goes fast. (D_2_)"

Here a Swedish doctor explained that migrants anticipated discrimination and therefore become defensive:

"A patient also somehow becomes defensive: “they never understand what I say, they do not treat me equally, I never get access to health care the same way as the others, I get no medicines and the others do”. So things like that. (D_1_)"

For that reason, migrants might according to her be perceived as aggressive or always unsatisfied leading to misunderstandings, mistrust and frustration on both parts.

#### Questioning the Swedish model of care

According to caregivers, the fact that migrants were used to meeting a doctor in the first instance when they sought care or were always prescribed drugs after each visit or were not actively involved in their care posed challenges to the care encounters. Caregivers expressed concern particularly over the mistrust of nurses who within the Swedish system have special competences and are the category of caregivers a patient meets first at a clinic. A specialist nurse of mixed background said:

"I am a nurse but most people expect to meet the doctor because this is what you want and it is difficult to explain, you have to explain that we have several different nurses who are specially educated to deal with diabetes or hypertonia. When in other countries you would meet a doctor, in Sweden the nurses are educated to do this. (N1)"

The mistrust of nurses was corroborated by a Swedish doctor who described how nurses complained to her about migrant patients not trusting them and requesting to meet doctors.

The caregivers also implied that migrants are unfamiliar with scheduled appointments and self-care. They tend to over-use health care and expected prescriptions after each encounter, often resulting in conflicts. According to this nurse (N1), “many people want to have prescriptions. This is where difficulties will come and this is where in some countries if you have money you can buy and you can go first instead of queuing.” A doctor explained further that:

"Folks are brought up or taught to go to the doctor when they are sick. One is a good parent if one goes to the doctor when the kid is sick. But in Sweden one is a bad parent if one goes to the hospital when the child has only a cold …. one is supposed to manage this at home. (D_1_)"

Another disturbing issue reported was the way information on medical conditions including, life-threatening or debilitating diseases was given and received. Caregivers expressed frustration over migrants’ overreaction to negative information and believed this practice shocked migrants. One doctor speculated that migrants were accustomed to caregivers who perhaps simply listened to symptoms and then prescribed treatment and therefore expected the same from Swedish care providers. In contrast, Swedish caregivers tend to disclose detailed medical information to patients also in an attempt to involve them in decision-making. Migrants were said to be used to authoritative doctors at home. They are therefore passive and expect the Swedish doctors would behave in the same way. One doctor said she had to adjust herself to meet migrants’ expectation of an authoritative doctor in the following way:

"I do become authoritarian; I become much more kind of the doctor within quotation marks. I do. I kind of maybe teach in a different way than what I would do otherwise. . . . I have something I called the best doctor’s voice; I really become a doctor and explain, I use it more. . . . I mean, it is a challenge in some way to sense, what or how much one shall talk, how much doctor one should be. (D_1_)"

Despite having migrant background, another doctor also deplored migrants’ passivity and high expectations on caregivers’ ability to cure illness. This was in contrast to the Swedish patient-centered care approach as indicated below:

"What I feel is that the Swedish patients would like to get involved with their illnesses, their treatments, they are well educated and they read on the internet and then make suggestions about treatment. How can we or can we do so? And then you plan together. But, some immigrants want the doctor to decide and instruct them to do this and that. Then it will be good. If you ask them: “shall we do so?” They say: ‘as you're the doctor you should decide.’ These are some of the things that I think differ. (D_2_)"

## Discussion

This study, based on interviews with a variety of caregivers and participant observation, has illuminated the tensions in caring for migrant patients who despite entering a new health care system, still make reference and are guided by the backgrounds and systems which have shaped their view of health and disease, their health seeking behaviors and their expectations from health care providers. In this way, the study is about the complex interaction of contextual factors in the old and the new health care systems or the environments described in the socio-ecological model. The caregivers have described the multiple factors contributing to the challenges they face in caring for migrant populations in Sweden as stemming from language difficulties; socio-cultural diversity and migrants’ difficulties to navigate a new health care system due especially to limited information, use of technology and a care approach that expects patients to participate actively in their care. From a socio-ecological model these factors appear at different levels of the system including at the individual, the interpersonal, institutional and macro-societal levels and as indicated influence health-seeking behavior, the provision of care and the interactions between migrants and caregivers. These findings echo those of others who have reported that migrant health care is the result of dynamic interactions of many factors including individual and societal characteristics [14,18,42,43].

The heterogeneity of migrant populations, comprising of people of varied social, cultural, religious and geographical backgrounds with different experiences was said to affect care delivery and use in ways highlighted in other studies [6,14,16,17]. Moreover, educational, religious and ethnic backgrounds, migration history and status, lifestyles, health and socioeconomic status are issues that come into play even when patients may come from one country and that caregivers need to consider in their delivery of care [14-17,19]. For instance, participants in this study described being challenged by low educated and very religious patients. Education was said to affect health literacy while religious beliefs interfered with care. Furthermore, some migrants, in particular women refused to be treated/seen by providers of opposite sex. However, two female participants explained they had never found it difficult to care for male patients. It could be discussed whether the fact that men are rule makers in more patriarchal structures make it easy for them to break the rule and meet female caregivers or whether old age is, as stressed by a caregiver in this study an advantage. Some researchers have therefore urged providers to be aware that every patient is an unique individual [6,7,9,13,44,45]. Meanwhile others warned about intergenerational and inter-ethnic variations within migrant groups [10,13,15]. The question is whether and how care providers can pay attention to the total context of the patient’s situation including their immigration status, literacy level, gender, family dynamics, religious beliefs and stress factors while providing care or they just apply ‘one-size-fits all approach’ while providing care.

Additionally, our findings suggest that the difference in social and cultural backgrounds between migrants and caregivers also challenges medical encounter and threaten acceptability and quality of care [6,11,13,16,17,45,46]. Participants in this study reported stereotypes and discriminatory attitudes among care providers that could perpetuate mistrust and generate dissatisfaction that could undermine the trust and cooperation necessary for a successful therapeutic relationship [6,8,11,13,44,47]. According to Ahlberg et al., migrants often experience tensions living in two worlds and negotiating their existence, within contexts and discourses promoting stereotypical representations, discrimination and marginalization [48]. For instance, Cuadra stressed in her report that the Swedish label ‘*Invandrare*’ (immigrant) itself is associated with other connotations than just being a social or administrative categorization of foreign born persons. She indicated that the term is criticized for the loaded meanings which focus on differences, and emphasize the ‘other’ (non-Swedish) in relation to modernity and a presumed homogenous Swedish culture [23]. Prejudiced assumptions by care providers may result in the limitation of services to migrants, poor access and acceptability [6]. Here the question is: what kind of knowledge do caregivers possess about migrants’ backgrounds and how do they get such knowledge.

Nevertheless, the most obvious challenge mentioned is language which prevents effective communication and creates frustrations for caregivers and patients with consequences for access, acceptability and quality of care as also described in other research [6,8,10,11,14,18,19,26,43,45]. Literature indicates that healthcare in general rely on dialogue. Language thus becomes the primary barrier at the interpersonal level leading to longer consultations and misunderstandings with increased risk for wrong diagnosis, inappropriate treatment and non-compliance [8,11,12,14-19,26,43,49]. Participants in this study expressed the difficulty for interpreters to translate medical terms correctly during care encounters, which has the potential risk of misleading both the patient and the caregiver. A recent survey showed that over 30 languages were used in a Swedish language school in Northern Sweden illustrating the growing diversity and challenges for care encounters [36,37]. Our study also suggests the societal and institutional influences in terms of policies and practices within the health care system. For instance, the Swedish policy of providing care, health information and hotline services in Swedish hinder access to and effective use of health information and services as well as the interaction between care providers and patients who cannot properly communicate or understand Swedish. This is particularly critical for individuals with low literacy skills, [6,8,10,11,19,26,37]. Fatahi et al. argued that the inclination among some migrant patients to prefer their mother tongue during medical encounters could also be in part due to the Swedish policy of offering language assistance [19,25]. However, our observations indicate that language is a substantial barrier to communication in cross-cultural encounters [6,10,26]. While some migrants might speak Swedish or English in their everyday life, it may be insufficient to read letters and leaflets or to understand medical jargon especially during medical encounters [14-16,37]. We observed a number of problems related to the use of interpreters including unprofessional conduct, inaccurate translations, patient discomfort, gender norms, medical confidentiality and use of relatives or friends for sensitive situations. For instance, the dual role of interpreters as both community members and professionals threaten confidentiality and negatively impacts on health care seeking behavior and care encounter. Similar challenges have been discussed in other studies in and outside Sweden [6,8,10,11,13,15,16,18,19,26,43,47,49]. Additionally, some studies emphasized issues of access to interpreters who are not integrated within the health care system, shortage of interpreters for particular languages as well as government pressures to economize as making the use of interpreters problematic [6,10,26]. The question is how issues related to language barrier and the use of interpreters should be addressed.

Apart from language, this study suggests that the use of distant communication technology (letters, telephone calls, e-mails, e-health information, and leaflets) at the institutional level also negatively affects access to available health services especially for migrants with low literacy skills. This has also been stressed by other authors [6,14,16,37,45]. Despite being modernized, the Swedish health care system may thus be inaccessible to migrants because patients are expected to communicate with caregivers via mail, web or phone. For example, the research participants in this study reported that migrants sought care as drop in patients because they were unable to use the booking system and this often resulted in conflicts as providers expected them to follow the rules and make prior appointments. In other cases, migrants were fined for not showing up as expected since; they could not call to cancel the appointment, even though an interpreter was already hired. The migrants’ unfamiliarity with the Swedish health care system and their inability to navigate through it is another illustration of challenges arising from institutional and societal influences. Migrants’ perceptions and expectations (required prescriptions, immediate care for non-emergency situations, and mistrust of nurses) about care and health care system were described to be rooted in their past experience in their home countries and this experience may be in contrast with the Swedish health care system and model of care. Furthermore, the Swedish model of care emphasizes patient autonomy through the patient-centered care approach in common use in most Western countries [12,17,20,22,45]. The assumption is that this model of care improves treatment choices, quality of care and outcomes through informed decision making. However, our findings indicate that involving migrants was difficult as migrants expected the doctor should know the diagnosis and should tell them what is best. Several authors argue too that the patient-centered approach may lead to significant ethical dilemmas and challenges because of misinterpretation arising from adhering to different explanatory models common in cross-cultural encounters [13,17,45,46]. Moreover, “truth telling”, or the disclosure of terminal diagnosis or other bad news can be a dilemma as the participants in this study described experiencing difficulty to reveal directly to the patient scaring diagnosis in the face of family and gender dynamics where migrant men may feel entitled as the primary authorities to receiving medical information concerning members of their families. Some researchers stated that in some countries, doctors are likely to withhold upsetting information from patients who seemed to prefer this withholding or a common practice is to reveal potentially upsetting information gradually and cautiously [17,44]. They, therefore, suggest that rather than communicating exclusively with a patient, health professionals should consider including a family spokesperson who is often regarded as able to cope with bad news and to determine whether or not to reveal the information to the patient and how best to do it [17,44].

Participants in this study described facing difficulties in understanding and communicating things to patients who hold different explanatory models about clinical realities. According to some authors, both patients’ and doctors’ behaviors and explanations about clinical realities are culture-specific. Thus, most of the problems in cross-cultural encounters result from hidden discrepancies between their views of clinical reality [6,13,17,46]. Kleiman et al. elucidated these discrepancies and argued that, clinicians within the Western medical paradigm often diagnose and treat diseases (malfunctioning of biological and psychophysiological processes in individuals) whereas patients suffer and seek help for illnesses (personal, interpersonal, and cultural reactions to disease or discomfort) [46]. The research participant in this study explained too that concerns about symptoms rather than disease prompted migrants to seek care or comply with treatment and preventive rules. This maybe an indication of a mismatch between providers’ and patients’ explanatory models and expectations and could explain low awareness about health promotion initiatives, the delay in seeking timely care for serious/chronic conditions such as diabetes or over-utilization of care services for minor/acute ailments described in this study. Other authors have similarly observed this to be the case [11-13,18,43,49]. Several authors have reported that these discrepancies lead to a sense of discrimination and dissatisfaction on the part of the patient and to frustration, misperceptions and prejudice against the patient on the part of the provider with negative consequences for the patient-provider relationship [11-14,17,18,43,44,46]. Expansive literature reported that patients’ explanatory model of illness often arouse expectations about treatment and healing process that might differ from that of clinicians and as reported in this study could create problems for clinical management [11-13,15,17,18,43-46]. Failure to take account of diverse explanatory models and expectations may compromise care and clinical management of diseases, especially for chronic and asymptomatic diseases [13,43,45,46]. Rather than contradicting patient’s explanatory model of clinical realities, providers should try to negotiate and find options that will be mutually acceptable to both and that incorporate patient’s beliefs, if necessary give clear clinical explanations or educate patients if their explanatory models are likely to interfere with appropriate/planned care.

Finally, our study illustrates that care providers face a number of challenges that have multiple layers manifested in language barrier, socio-cultural diversity and the difficulty for migrants to navigate the Swedish health care system. As stressed by Liburd & Sniezek, the synergistic relationship among the multiple and complex layers represented in the socio-ecological model make it difficult to identify any single component or factor embedded within the model as the driving factor [29]. This calls for actions and policies that encompass all levels and that are interactive and two-sided. For instance, it is unrealistic to expect migrants to change behavior if caregivers do not change their own and if barriers at other levels are not addressed.

### Study strengths and limitations

This study has strengths and weaknesses as is common in qualitative research. Ten informants from one area, all interviewed by a single investigator with migrant background, with a potential desire on respondents’ part to appear morally upright, potentially threaten the credibility and transferability of the study. Nevertheless, the findings of an ID inquiry are not a list of isolated themes, but a synthesis of the main themes and patterns of the phenomena that experts in the area will acknowledge as persuasive [31-33]. Moreover, during data collection and analysis, we relied on accepted principles of trustworthiness to allow the reader of the research report evaluate the relevance of data on which findings were based, the logic by which the conclusions were drawn, and the degree to which the interpretations reflect a coherent and grounded conclusion [32-35]. To enhance credibility, we used different kind of data sources to compensate for their shortcomings and exploit their benefits to understand and verify particular details that were provided. In addition, we used a wide range of participants and different settings so that we could check information across informants. All these provided triangulation, reduced investigator bias and contribute to the trustworthiness of generated findings [32,34,35]. Furthermore, the main investigator had debriefing sessions with other members of the research team (co-authors) who reviewed the interview guide, style and participated in the analysis process during the study itself. Information about the background, experience and qualifications of the main investigator has also been provided [35]. Moreover, the findings were presented and discussed in seminars with other care providers caring for migrants in other parts of Sweden. To address dependability issues, we have reported the research design and its implementation in details and followed the operational details of data collection and analysis.

Although, purposive sampling does not confer transferability, it does, however, provide in-depth information from different individuals representing valuable perspectives, which also strengthen the findings. For instance, while doctors and nurses appeared to emphasize a more allopathic perspective, the social workers showed more empathy and understanding about migrants’ difficulty to adapt to the new environment, which emphasize the need for further exploration. In addition, public health officers described other aspects of the challenges that are uncommon in the clinical setting especially health promotion. However, the migrant doctor’s acculturation made him look at migrant patients with more “Swedish eyes”. Without offering firm assurances for transferability, we believe that most of our findings are of a more general nature and we do also note that they concur with previous studies on challenges in cross-cultural care encounters [6-8,10,12,17,18,26,45,47,49]. Although we can say that we have a rich description that could enable transferability, there is a need for caution when applying these findings to other cross-cultural care contexts due to differences in health care policies and migration context. Finally, although providers discussed perceived challenges for migrants, the findings do not represent the personal experiences, opinions or feelings of migrants. Nevertheless, this study is part of a research project on challenges and opportunities in HIV/AIDS and Tuberculosis care among migrants and is based on findings from a survey study with migrant students [36,37]. Research on migrants’ and interpreters’ experiences with the health care system is planned as part of this project to better understand their needs and how to improve the delivery and receipt of care.

## Conclusions

Sweden is an increasingly diverse country and in this study, we illuminate the complex and interlinked issues about caring for migrants. Although the study was carried out within the health care setting, these issues span beyond the health care system and encompass other public services. The caregivers described migrants as very heterogeneous populations comprising people of various social, cultural, linguistic, religious and geographical backgrounds with different experiences all which affect care delivery and use. These findings concur with national and international literature on cross-cultural care. The article describes common challenges in cross-cultural encounters within the Swedish context. The challenges described are interconnected at individual, institutional and societal levels highlighting the need for multifaceted approaches to improve the delivery and receipt of care. The policy implications of these findings are discussed in relation to the need: to adapt care to the individual needs, to train interpreters or bicultural staff to act as interpreters, to include information regarding migrants’ literacy profiles and language preferences into the identification section of personal medical files, to translate key documents, hotline messages, information about the health care system and health education material in formats and languages that are accessible and acceptable to migrants and to improve migrants’ health literacy and effective use of services through community based educational outreach programs.

## Competing interests

The authors declared no potential conflicts of interest with respect to the research, authorship, and/or publication of this article.

## Authors’ contributions

FKNK conceived of the study, and participated in its design, and carried out interview and observation studies and analysis, and drafted the manuscript. BMA participated in the design of the study, and analysis, and coordination and helped to draft the manuscript. AKH and CA participated in the analysis and helped to draft the manuscript. All authors read and approved the final manuscript.

**AKH** is an associate professor of Public Health at the Umeå University Department of Public Health and Clinical Medicine Division of Epidemiology and Global Health in Umeå, Sweden.

**CA** is a senior clinician and professor at the Umeå University Department of Clinical Microbiology Division of Infectious Diseases in Umeå, Sweden.

**BMA** is a professor of International Health (Sociology background) at the Uppsala University Department of Women’s and Child Health in Uppsala and Head of research at the Skaraborg Institute of Research and Development in Skövde, Sweden.

## Authors’ information

**FKNK** is a medical doctor and trained interpreter with Master’s degree in Public Health and currently a doctoral student at the Umeå University Department of Public Health and Clinical Medicine Division of Epidemiology and Global Health in Umeå, Sweden.

## Pre-publication history

The pre-publication history for this paper can be accessed here:

http://www.biomedcentral.com/1472-6963/12/433/prepub

## References

[B1] International Organization for Migration World Migration Report 2010. The future of Migration: Building Capacities for Changes World Migration Report 2010 IOM

[B2] United Nations High Commissioner for Refugees UNHCR2009 Report. 2009 Global Trends: refugees, Asylum-seekers, Returnees, Internally displaced and Stateless Persons 2010 UNHCR

[B3] Carballo M Mboup M International migration and health 2005 International Centre for Migration and Health http://www.iom.int/jahia/webdav/site/myjahiasite/shared/shared/mainsite/policy_and_research/gcim/tp/TP13.pdf

[B4] Statistics Sweden Description of the Population in Sweden 2008 Befolkningsstatistik Population statistics 2009 http://www.scb.se/statistik/_publikationer/BE0101_2008A01_BR_BE0109TEXT.pdfStatistcs

[B5] Statistics Sweden The future population of Sweden 2009–2060 Demographic Reports 2009 Statistics Sweden

[B6] Akhavan S Karlsen S Practitioner and Client Explanations for Disparities in Heatlh Care Use Between Migrant and Non-migrant Groups in Sweden: A Qualitative Study J Immigr Minor Health 2012 10.1007/s10903-012-9581-y 22323124

[B7] Hultsjö S Berterö C Arvidsson H Hjelm K Core components in the care of immigrants with psychoses: A Delphi survey of patients, families, and health-care staff Int J Ment Health Nurs 2011 20 174 184 10.1111/j.1447-0349.2010.00720.x 21352448

[B8] Hultsjö S Hjelm K Immigrants in emergency care: Swedish health care staff's experiences Int Nurs Rev 2005 52 276 285 10.1111/j.1466-7657.2005.00418.x 16238724

[B9] Jensen NK Nielsen SS Krasnik A Expert opinion on "best practices" in the delivery of health care services to immigrants in Denmark Dan Med Bull 2010 57 8 A4170 20682134

[B10] Jirwe M Gerrish K Emami A Student nurses' experiences of communication in cross-cultural care encounters Scand J Caring Sci 2010 24 436 444 10.1111/j.1471-6712.2009.00733.x 20233352

[B11] Priebe S Sandhu S Dias S Gaddini A Greacen T Ioannidis E Kluge U Krasnik A Lamkaddem M Lorant V Good Practice in health care for migrants: Views and experiences of care professionals in 16 European countries BMC Public Health 2011 11 187 10.1186/1471-2458-11-187 21439059 PMC3071322

[B12] Schouten BC Meeuwesen L Cultural differences in medical communication: A review of the literature Patient Educ Couns 2006 64 21 34 10.1016/j.pec.2005.11.014 16427760

[B13] Shaw SJ Huebner C Armin J Orzech K Vivian J The Role of Culture in Health Literacy and Chronic Disease Screening and Management J Immigrant Minority Health 2009 11 460 467 10.1007/s10903-008-9135-5 18379877

[B14] Szczepura A Access to health care for ethnic minority populations Postgrad Med J 2005 81 141 147 10.1136/pgmj.2004.026237 15749788 PMC1743229

[B15] Newbold KB Willinsky J Providing family planning and reproductive healthcare to Canadian immigrants: perceptions of healthcare providers Cult Health Sex 2009 11 4 369 382 10.1080/13691050802710642 19242836

[B16] Zanchetta MS Poureslami IM Health Literacy within the Reality of Immigrants' Culture and Language Can J Public Health 2006 97 2 526 530 10.1007/BF03405370 PMC6975770 16805158

[B17] Betancourt JR Green AR Carrillo JE The challenges of cross-cultural healthcare – diversity, ethics, and the medical encounter Bioethics Forum 2000 16 3 27 32 12528728

[B18] Shtarkshall RA Barnesan F Feldman BS A Socio-ecological analysis of Ethiopian immigrants' interactions with the Israeli healthcare system and its policy and services implications Ethn Health 2009 14 5 459 478 10.1080/13557850902890522 19488916

[B19] Fatahi N Hellström M Skott C Mattsson B General practitioners' views on consultations with interpreters: A triad situation with complex issues Scand J Prim Health Care 2008 26 1 40 45 10.1080/02813430701877633 18297562 PMC3406627

[B20] The Swedish Ministry of Health and Social Affairs Health and medical care in Sweden 2007 http://www.sweden.gov.se/content/1/c6/08/60/40/982480dd.pdf.

[B21] Cuadra CB Policies on Health Care for Undocumented Migrants in EU27- Country report Sweden Health care in NOWHERELAND- Improving services for undocumented migrants i the EU 2010 Malmö university, Malmö

[B22] The Swedish National Board of Health and Welfare Din skyldighet att informera och göra patienten delaktig - Handbok för vårdgivare, chefer och personal 2012 Socialstyrelsen http://www.socialstyrelsen.se/lists/artikelkatalog/attachments/18552/2012-1-5.pdf

[B23] MIGHEALTHNET State of the Art Report (SOAR) Sweden http://www.mighealth.net/se/images/9/93/state_of_the_art_Report_%28SOAR%29_Sweden_09.pdf

[B24] The Swedish National Board of Health and Welfare Socialstyrelsens föreskrifter och allmänna råd om hälsoundersökning av asylsökande m.fl Volume SOSFS 2011:11 (M) 2011 The Swedish National Board of Health and Welfare, Västerås

[B25] The Swedish Riksdag Förvaltningslag (1986:223) Volume SFS 1986:223 1986 http://www.riksdagen.se/sv/Dokument-Lagar/Lagar/Svenskforfattningssamling/_sfs-1986-223/

[B26] Hadziabdic E Heikkilä K Albin B Hjelm K Problems and consequences in the use of professional interpreters: qualitative analysis of incidents from primary healthcare Nurs Inq 2011 18 3 253 261 http://onlinelibrary.wiley.com/doi/10.1111/j.1440-1800.2011.00542.x/pdf 10.1111/j.1440-1800.2011.00542.x 21790876

[B27] Betancourt JR Green AR Carrillo JE Cultural competence in health care: emerging frameworks and practical approaches Field Report 2002 The Commonwealth Fund

[B28] Bronfenbrenner U Ecological system theories Annals of Child Development 1989 6 187 249

[B29] Liburd LC Sniezek JE Changing Times: new possibilities for community health and well being Prevention of Chronic Disease 2007 http://www.ncbi.nlm.nih.gov/pmc/articles/PMC1955418/pdf/PCD43A73.pdf PMC1955418 17572977

[B30] Yakushko O Chronister KM Immigrant Women and Counseling: The Invisible Others Journal of Counseling & Development 2005 83 292 298 10.1002/j.1556-6678.2005.tb00346.x

[B31] Hunt MR Strenghts and Challenges in the Use of Interpretive Description: Reflections Arising From a Study of the Moral Experience of Health Professionals in Humanitarian Work Qual Health Res 2009 19 9 1284 1292 10.1177/1049732309344612 19690208

[B32] Thorne S Interpretive Description 2008 Left Coast Press edition, Walnut Creek

[B33] Thorne S Kirkham SR O'Flynn-Magee K The Analytic Challenge in Interpretive Description Int J Qual Methods 2004 3 1

[B34] Patton MQ Enhancing the Quality and Credibilty of Qualitative Analysis Health Serv Res 1999 34 5 1189 1208 10591279 PMC1089059

[B35] Shenton AK Strategies for ensuring trustworthiness in qualitative research projects Educ Info 2004 22 2 63 75

[B36] Nkulu FK Hurtig A-K Ahlm C Krantz I Fear of Deportation May Limit Legal Immigrants’ Access to HIV/AIDS-Related Care: A Survey of Swedish Language School Students in Northern Sweden J Immigr Minor Health 2012 14 39 47 10.1007/s10903-011-9509-y 21814777 PMC3256311

[B37] Nkulu FK Hurtig A-K Ahlm C Krantz3 I Screening migrants for tuberculosis - a missed opportunity for improving knowledge and attitudes in high-risk groups: A cross-sectional study of Swedish-language students in Umeå Sweden. BMC Public Health 2010 10 349 10.1186/1471-2458-10-349 PMC2905331 20565732

[B38] Dwyer SC Buckle JL The Space Between:On Being an Insider-Outsider in Qualitative Research Int J Qual Methods 2009 8 1 54 63

[B39] Braun V Clarke V Using thematic analysis in psychology Qual Res Psychol 2006 3 2 77 101 10.1191/1478088706qp063oa

[B40] Green J Willis K Hughes E Small R Welch N Gibbs L Daly J Generating best evidence from qualitative research: the role of data analysis Aust N Z J Public Health 2007 31 6 545 550 10.1111/j.1753-6405.2007.00141.x 18081575

[B41] World Medical Association Declaration of Helsinki -Ethical Principles for Medical Research Involving Human Subjects 2008 http://www.wma.net/en/30publications/10policies/b3/17c.pdf

[B42] Choi JY Contextual effects on health care access among immigrants: Lessons from three ethnic communities in Hawaii Soc Sci Med 2009 69 1261 1271 10.1016/j.socscimed.2009.08.001 19720437

[B43] Pavlish CL Noor S Brandt J Somali immigrant women and the American health care system: Discordant beliefs, divergent expectations, and silent worries Soc Sci Med 2010 71 353 361 10.1016/j.socscimed.2010.04.010 20494500 PMC2893335

[B44] Aboul-Enein BH Aboul-Enein FH The Cultural Gap Delivering Health Care Services To Arab American Populations in the United States J Cult Divers 2010 17 1 20 23 20397570

[B45] Jayadevappa R Chhatre S Patient Centered Care - A Conceptual Model and Review of the State of the Art The Open Health Services and Policy Journal 2011 4 15 25 10.2174/1874924001104010015

[B46] Kleiman A Eisenberg L Good B Culture, Illness and Care: Clinical Lessons from Anthropologic and Cross-Cultural Research Journal of Lifelong Learning in Psychiatry 2006 IV 1 140 149 10.7326/0003-4819-88-2-251 626456

[B47] Caroll J Epstein R Fiscella K Gipson T Volpe E Jean-Pierre P Caring for Somali women: Implications for clinician-patient communication Patient Education and Counselling 2007 66 337 345 10.1016/j.pec.2007.01.008 PMC3298771 17337152

[B48] Ahlberg BM Lindmark G Krantz I Warsame M ‘It’s only a tradition’: making sense of eradication interventions and the persistence of female ‘circumcision’ within a Swedish context Critical Social Policy 2004 24 50 50 78

[B49] Abbott S Riga M Delivering Services to the Bangladeshi community: the views of healthcare professionals in East London Public Health 2007 121 12 935 941 10.1016/j.puhe.2007.04.014 17655892

